# Bronchoscopist-Directed Continuous Flow Propofol Based Analgosedation during Flexible Interventional Bronchoscopy and EBUS

**DOI:** 10.3390/jcm12134223

**Published:** 2023-06-22

**Authors:** Georg Evers, Michael Mohr, Lena Sprakel, Jule Galonska, Dennis Görlich, Arik Bernard Schulze

**Affiliations:** 1 Department of Medicine A, Hematology, Oncology and Pulmonary Medicine, University Hospital Münster, 48149 Münster, Germany; michael.mohr@ukmuenster.de (M.M.); lena.sprakel@ukmuenster.de (L.S.); jule.galonska@uni-muenster.de (J.G.); 2 Institute of Biostatistics and Clinical Research, Westfälische Wilhelms-University Münster, 48149 Münster, Germany; dennis.goerlich@ukmuenster.de

**Keywords:** deep analgosedation, EBUS, interventional bronchoscopy, propofol

## Abstract

Sedation techniques in interventional flexible bronchoscopy and endobronchial ultrasound-guided transbronchial-needle aspiration (EBUS-TBNA) are inconsistent and the evidence for required general anesthesia under full anesthesiologic involvement is scarce. Moreover, we faced the challenge of providing bronchoscopic care with limited personnel. Hence, we retrospectively identified 513 patients that underwent flexible interventional bronchoscopy and/or EBUS-TBNA out of our institution between January 2020 and August 2022 to evaluate our deep analgosedation approach based on pethidine/meperidine bolus plus continuous flow adjusted propofol, the bronchoscopist-directed continuous flow propofol based analgosedation (BDcfP) in a two-personnel setting. Consequently, 502 out of 513 patients received BDcfP for analgosedation. We identified cardiovascular comorbidities, chronic obstructive pulmonary disease, and arterial hypertension as risk factors for periprocedural hypotension. Propofol flow rate did not correlate with hypotension. Theodrenaline and cafedrine might be used to treat periprocedural hypotension. Moreover, midazolam might be used to support the sedative effect. In conclusion, BDcfP is a safe and feasible sedative approach during interventional flexible bronchoscopy and EBUS-TBNA. In general, after the implementation of safety measures, EBUS-TBNA and interventional flexible bronchoscopy via BDcfP might safely be performed even with limited personnel.

## 1. Introduction

Guidelines on the performance of flexible interventional bronchoscopy and endoscopic ultrasound (EBUS) guided transbronchial needle aspiration (TBNA) primarily include requirements and expertise in the process itself [1,2,3,4]. However, data on periprocedural sedation are lacking evidence. Hence, recent guideline recommendations have been predominantly based on expert opinions and sedation follows guidelines of gastrointestinal endoscopy [5] and recommendations on sedation in flexible bronchoscopy [6]. Here, deep analgosedation may be used during interventional flexible bronchoscopy and EBUS-TBNA [1] but, largely, EBUS is performed under general anesthesia including analgesia, intravenous sedation as well as a muscle relaxant with simultaneously controlled ventilation via laryngeal mask airway, endotracheal tube [7], or rigid bronchoscopy.

In times of limited financial and personnel resources, interventional bronchoscopy requires pre-planned appropriate temporal frames. Regarding this, rigid bronchoscopy implicitly requires full teams of anesthesiology and interventional bronchology to perform bronchoscopy under general anesthesia as well as controlled jet ventilation [8], frequently performed in an operating room. Flexible interventional bronchoscopy as well as EBUS-TBNA, however, might be performed via deep analgosedation in an endoscopy suite by one or more bronchoscopists, supported by the endoscopy nurse [7].

So far, in Germany, laws and strict rules for sedation during endoscopy have not yet been established. However, the German Respiratory Society has set up recommendations for sedation during flexible bronchoscopy. As a consequence, non-anesthesiological guided propofol use in endoscopy is generally permitted [6] and largely performed [9]. Before the procedure, the S3-guideline for flexible bronchoscopy proposes risk assessment with respect to the expected duration of the intervention as well as the patient’s comorbidities, e.g., by classifying the patients according to the proposals of the American Society of Anesthesiologists (ASA) [10]. Moreover, after sedation initiation by a trained physicist, continuous supervision of the sedation may be performed by a specially trained nurse as the so-called nurse-administered propofol sedation (NAPS) [6].

At our institution, pethidine/meperidine plus propofol [11] is the most commonly used analgosedation and can be supported by midazolam to reduce propofol doses [5,12]. Endoscopist-directed propofol sedation (EDP) has been evaluated as a reasonable and safe sedative approach in gastrointestinal endoscopy [13,14]. However, data on its application in flexible interventional bronchoscopy and EBUS are rare. A small study of 31 patients receiving continuous flow propofol sedation stated a practical, effective, and safe sedation technique [15]. Moreover, a Swiss randomized noninferiority trial compared bolus applications of propofol to a continuous infusion protocol, resulting in comparable safety profiles with higher propofol doses for the continuous flow protocol [16].

A widely used alternative is the combination of ketamine plus midazolam [17,18], which, in our case, was predominantly used in patients with a high risk of periprocedural hypotension, such as high-grade congestive heart failure.

At our institution, we faced the challenge of providing respiratory and bronchoscopic care with very limited personnel. Thus, we used this retrospective analysis to document and evaluate real-world data on the reasonable sedative pethidine/meperidine and continuous flow propofol approach during flexible interventional bronchoscopy and EBUS-TBNA at an endoscopy suite with limited personnel. As a part of the non-anesthesiological application of propofol (NAAP) [5,13], the term endoscopist-directed propofol sedation (EDP) [14] was modified into bronchoscopist-directed continuous flow propofol based analgosedation (BDcfP), in resemblance to the study of Chrissian and Bedi [15].

## 2. Material and Methods

### 2.1. Study Collective

Retrospectively, we identified *n* = 513 patients that underwent interventional flexible bronchoscopy and/or EBUS-TBNA at the department of pulmonary medicine at the University Hospital Münster between January 2020 and August 2022. To reduce confounding effects, all examined patients during this period were included in this real-world retrospective data analysis. Patients gave informed consent to the pre-planned procedure between 1 to 14 days before the intervention.

### 2.2. Safety Measurements and Procedure

After obtaining informed consent and being risk-assessed via ASA score [10], each patient underwent a spirometry or body plethysmography to screen for restrictive or obstructive pulmonary disorders, if possible. Moreover, an arterialized capillary blood gas analysis was conducted to gather information regarding impaired gas exchange. Laboratory analyses were performed at a maximum of 7 days before intervention and included a hemogram as well as parameters of partial thromboplastin time (PTT) and international normalized ratio (INR). Besides a lack of sufficient data regarding interventional bronchoscopy [2,19], site-internal standards required documented platelets above 80,000/µL. Patients with platelets between 50,000/µL and 80,000/µL were discussed by at least two trained bronchoscopists with respect to the anticipated risk of bleeding against the background of the planned intervention. Below the platelet cutoff of 50,000/µL, a transfusion was performed prior to bronchoscopic intervention. Phenprocoumon and warfarin were usually bridged by lower molecular weight heparins, which in turn were paused 24 h before intervention. In this case, coagulation diagnostic was repeatedly obtained at the day of the intervention to document an INR below 1.5 and a PTT below 45 s. Novel oral anticoagulants (NOAC) were paused at least 24 h before intervention. Dual antiplatelet therapies (i.e., 100 mg acetylsalicylic acid [ASS] plus 75 mg clopidogrel, or 100 mg ASS plus 10 mg prasugrel, or 100 mg ASS plus 2 × 90 mg ticagrelor) were reduced to 100 mg ASS monotherapy five to seven days before the intervention [20,21], if allowed.

Each intervention was performed by at least one experienced and “Advanced Cardiac Life Support” (ACLS)-trained bronchoscopist and at least one experienced endoscopy-trained nurse. A second experienced and ACLS-trained bronchoscopist was pre-informed, working facing the bronchoscopy suite and attainable via alarm bell to reach the suite in a maximum of 15–20 s at any time. Additional personnel in training were eventually involved in sedation and bronchoscopy. In cases of pre-identified severe comorbidities (e.g., ASA score 4), special sedation techniques, or complex procedures, a second trained bronchologist was situated at the endoscopy suite, controlling the procedure, and supporting in analgosedation and patient care.

Before initiation of analgosedation, patients were provided with a 20-gauge peripheral intravenous catheter. Moreover, patients obtained lidocaine 2% spray orally to anesthetically pretreat the posterior pharyngeal wall, and pre-oxygenation over a nasal cannula was performed via an oxygen flow of 2–4 L/min. Before the initiation of analgosedation, patients received a bite block to provide an orifice for safe bronchoscopy and oral intubation.

Before, during, and after analgosedation, patients were monitored with continuous electrocardiogram documentation, automated, two-minute repeated non-invasive systolic (*SBP*) and diastolic blood pressure (*DBP*) measurements, continuous oxygen saturation measurement, and in case of pulmonary comorbidities, non-invasive percutaneous capnography until full conscious recovery. Mean blood pressure (*MBP*) was subsequently calculated via the following formula.

(1) $$MBP=DBP+\frac{1}{3}\;\left(SBP-DBP\right)$$

Analgosedation was predominantly performed intravenously with a fix-dose of 50 mg pethidine/meperidine accompanied by an initial bolus of 0.5 mg/kg propofol and followed by a continuous flow rate of 0.5 mL/kg 1% propofol. To achieve an adequate deep sedative effect, boluses of 10–20 mg propofol were administered, and the continuous flow rate was moderately increased until the patient was irresponsive to the lid closure reflex. Overall, the propofol management was performed largely in line with its administration performed by Lo et al. [22]. Occasionally, high continuous flow rates of propofol were supported by intravenous midazolam boluses of 1 to 2.5 mg to support the sedative effect [5]. Pethidine/meperidine was omitted in case of pre-existing opioid therapy, such as oral hydromorphone, tramadol, or higher-dosed tilidine.

For patients with non-catecholamine-dependent, severe congestive heart failure, analgosedation was performed with ketamine and midazolam [11,17,18,23,24] by a second experienced bronchologist.

After application of a maximum of 4.5 µg/kg lidocaine, 2% local anesthesia to the larynx, trachea and bronchi via the bronchoscope, a xylocaine gel pretreated Ø 8.5 mm double lumen tube (“bronchoflex” pattern, e.g., mediland GmbH, D-73635 Rudersberg, Germany, Ref. ML-1092085) was released over the scope and set unblocked at tracheal level. The primary lumen was then used for interventional bronchoscopy and endobronchial ultrasound, while the second lumen was used for oxygen supplementation at a flow rate to ensure a stable saturation of at least 90% in the spontaneously breathing patient. In case of insufficient saturation or insufficient spontaneous breathing, temporary supportive manual ventilation via a ventilation bag was performed after blocking the tube.

After the establishment of safe airways, the interventional flexible bronchoscopy as well as the EBUS-TBNA was performed as described in various guidelines [1,2,3].

Adequate sedation depth in the spontaneous breathing patient was continuously controlled via monitoring of a normofrequent heart rate (i.e., 60/min to 90/min), as well as repeated monitoring of normotensive blood pressure (i.e., 90/60 mmHg to 140/80 mmHg), the responsiveness to lid closure reflex and an absent cough reflex.

Anticipated depression of blood pressure was treated with continuous intravenous electrolyte solutions (e.g., B Braun Sterofundin ISO 500 mL, B. Braun SE, D-34209 Melsungen, Germany) and/or needs-based treated with intravenous application of 100–200 mg of cafedrine plus 5–10 mg theodrenaline. Occasionally, three patients received pre-planned moderate doses of continuous flow norepinephrine and one patient received 1 mg of epinephrine.

Emergency drugs such as flumazenil (0.1 mg/mL), naloxone (0.4 mg/mL), epinephrine (0.1 mg/mL), atropine (1.0 mg/mL) [25], prednisone (10 mg/mL), metoprolol (1 mg/mL), amiodarone (50 mg/mL) and ajmaline (5 mg/mL) were easily accessible within the endoscopy suite.

## 3. Statistical Analysis

To describe the cohort, we used mean, standard deviation (SD), median, interquartile range (Q_1_–Q_3_), 95 percent confidence interval (95% CI) as well as raw count and frequencies. Two-fold associations between categorical variables were analyzed via Fisher’s exact test or Chi-square test, if applicable. Paired continuous and ordinal variables were tested using either paired *t*-test or Friedman test, depending on the normality of the data. Unpaired continuous and ordinal variables were tested using either an unpaired *t*-test or the Mann–Whitney U test, depending on the normality of the data. Unpaired continuous and ordinal variables of more than two discriminators were tested using either One-Way ANOVA or Kruskal–Wallis test, depending on the normality of the data. The correlation coefficient r was calculated via two-tailed Spearman’s rank correlation. 

Data collection, calculations as well as chart generation were performed using IBM^®^ SPSS^®^ Statistics Version 29 (released 2022, IBM Corp., Armonk, NY, USA). The local significance level was set to 0.05. Due to the explorative character of the analysis, an adjustment to multiplicity was not determined

## 4. Results

### 4.1. Baseline Characteristics

The baseline characteristics of the evaluated cohort can be found in Table 1. A total of 513 patients were documented of whom one-third were female. The average age of the cohort was 60.5 years. Pulmonary comorbidities including chronic obstructive pulmonary disease, bronchial asthma, and pre-diagnosed obstructive sleep apnea were apparent in 19.3% of the patients. Moreover, cardiovascular comorbidities including coronary artery disease, congestive heart failure, and cerebral or peripheral obstructive artery disease were found in 17.9% of the evaluated cohort. Other than that, arterial hypertension was the most frequent comorbidity (39.0%), but diabetes mellitus (12.7%) and renal insufficiency (7.0%) were also documented in a relevant number of patients. With respect to ASA risk assessment, the mean ASA score was 2.8 (±0.5), and the median ASA score was 3 (Q_1_–Q_3_ 3–3). The proportionate distribution can be found in Table 1.

### 4.2. Procedural Characteristics and Use of Sedation

A second trained bronchoscopist was on site in 29.8% of the cases, mostly due to a predefined impaired patient condition (e.g., ASA score 4), the use of midazolam and/or ketamine (*p* < 0.001) as well as the use of the cryo probe (*p* < 0.001), or complex procedures, including fluoroscopy (*p* < 0.001), and navigation bronchoscopy (*p* < 0.001).

Including pre- and post-interventional nursing care, the average procedure time in our cohort was 92.2 (±29.6) minutes. By reduction of pre-interventional patient positioning and monitoring, as well as post-interventional observation, the actual mean interventional phase itself lasted 55.7 (±24.6) minutes (see Table 2).

A total of 493 cases (96.1%) received the combination of pethidine/meperidine plus propofol, and 9 cases (1.8%) were sedated with propofol alone due to preexisting oral opioid therapy. Propofol-supportive midazolam treatment was used in 37 out of 502 cases.

Ketamine plus midazolam sedation, however, was used in only 10 cases (1.9%), while two patients received additional ketamine to previously applied propofol, pethidine/meperidine, and midazolam due to inadequate deep sedation of the previously given medication and an insufficient high continuous flow rate of propofol.

As pethidine/meperidine was used as a fix-dose of 50 mg, slight deviances in Table 2 can be explained due to a single second opioid treatment for dyspnea in a palliative care patient after the intervention, as no other opioid was available at our endoscopy suite.

The mean propofol 1% perfusor flow rate was 62.9 (±36.6) mL/h (i.e., 10.5 [±6.1] mg/min), its median value was 54.5 (Q_1_–Q_3_ 39–75) mL/h. The absolute propofol doses used can be found in Table 2, complemented by already mentioned doses of pethidine/meperidine and midazolam.

**Figure 1:**
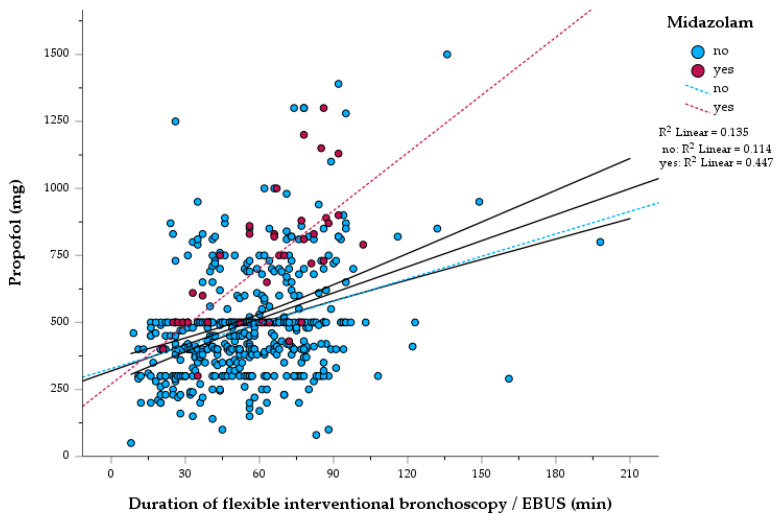
Scatter dot plot: Absolute propofol dose and duration of intervention of *n* = 502 BDcfP sedated patients. Scatter dot plot depicting the propofol dose over time, blue dots: without midazolam, red dots: with midazolam. The black line indicates simple linear regression with 95% confidence intervals of propofol doses over time. The red dotted line indicates the simple linear regression of propofol doses co-administered with midazolam. The blue dotted line indicates the simple linear regression of propofol doses if sedation was performed without additional midazolam. Spearman rank correlation coefficient: *r* = 0.345 (*p* < 0.001).

Figure 1 depicts the periprocedural absolute propofol doses over time. Of interest, the mean propofol dose in 502 patients was 500.3 (±217.0) mg and the interquartile range was between 370 to 565 mg at a median propofol dose of 480 mg. Propofol doses correlated positively with the procedure time (*r* = 0.345, *p* < 0.001, Figure 1) but additive midazolam treatment was not associated with higher propofol doses (*r* = 0.275, *p* < 0.001, Figure 1) or longer procedure time (*r* = 0.098, *p* = 0.028). 

EBUS/interventional flexible bronchoscopy yielded a diagnosis in 60.2% of the patients. On the contrary, in 39.8% of the patients, malignancy or specific inflammation was ruled out. Histopathological and cytological diagnoses included non-small cell lung cancer (NSCLC) in 110 patients (21.4%) and small cell lung cancer (SCLC) plus large cell neuroendocrine lung cancer (LCNEC) in another 43 patients (8.4%). Other malignant diseases were found in 52 cases (10.1%). The most prominent benign alteration was epithelioid cell granulomatosis as a specific inflammation pattern for sarcoidosis in 61 patients (11.9%). Other pulmonary or lymphonodal manifestations of rheumatic diseases (1.6%) and other interstitial lung diseases (1.4%) were rarely found. Infectious diseases were diagnosed in 13 patients, another 15 patients were diagnosed with lymphonodal anthracosis (see Table 2).

### 4.3. Performed Interventions and Complications

Next to bronchial washing and bronchoalveolar lavage (BAL), each patient received at least an EBUS, forceps biopsy, cryo biopsy, or brush cytology, eventually supplemented by fluoroscopy, argon plasma coagulation/high-frequency electricity or navigation bronchoscopy. Of note, the use of high-frequency electricity was mostly due to a high-frequency needle-knife prior to the cryo-biopsy of the mediastinal lymph nodes, as proposed by Zhang et al. [26].

On average, 5.8 needle aspirates were gathered per patient. Yet, the total amount of TBNAs ranged from 0 to 18 aspirates per patient. The most prominently assessed lymph node was on position 7, followed by 11R, 10R, 4R, and 11L (see Table 3).

With respect to complications, bleeding occurred in 13.6% of the cases. While in most cases, minor bleeding was self-limited after bronchoscopic suction, in about one-third of the bleeding complications, diluted adrenaline, oxymetazoline, or cold saline was applied and—after the termination of bleeding—eventually supported by argon-plasma coagulation (APC) (see Table 3). A bronchial obstruction, requiring steroid treatment, occurred in 33 of 513 patients of the cases. Moreover, agitation under BDcfP was present in six patients, requiring additional midazolam treatment. Another three patients required anti-hypertensive treatment (i.e., urapidil or metoprolole intravenously) despite the unresponsiveness of the lid closure reflex and an absent cough reflex.

### 4.4. Management of Vital Parameters during the Intervention

Of the patients, 4.9% (*n* = 25) did not receive any crystalloid infusion therapy. The other patients received Sterofundin ISO at a mean amount of 488.4 (±177.5) mL. The median crystalloid solution amount was 500 (Q_1_–Q_3_: 500–500) mL.

Periprocedural average and median vital parameters of all *n* = 502 continuous flow propofol sedated patients can be found in Table 4. Average heart frequency ranged from 78 (±16)/min before the intervention to 75 (±14)/min after the intervention. Moreover, oxygen saturation dropped from a pre-interventional average of 97.0 (±2.4)% to 95.8 (±5.4)% during the intervention and back again to 96.7 (±1.9)% afterward.

During the procedure, average blood pressure dropped from 135/77 mmHg to 98/62 mmHg (*p* < 0.001). With the end of the sedative effect, the blood pressure rose back to 115/69 mmHg, still showing a significantly reduced height compared with the initial value (*p* < 0.001) (see Table 4).

**Figure 2:**
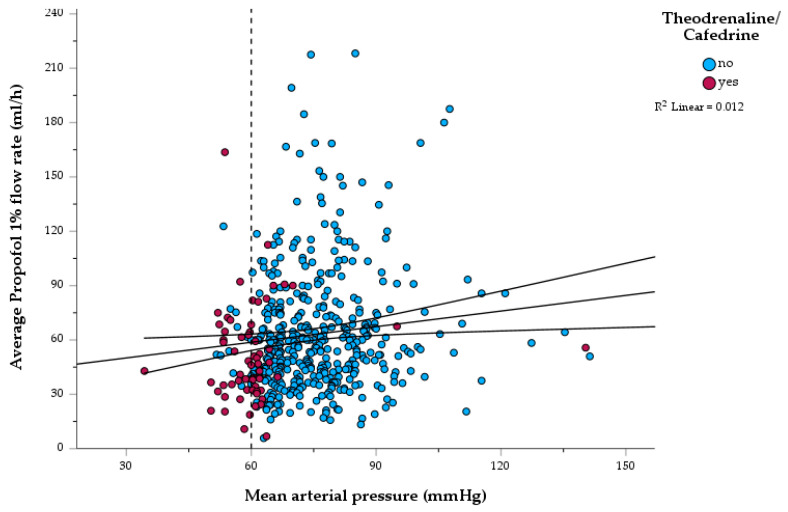
Scatter dot plot: Average Propofol 1% flow rate and periprocedural mean arterial pressure of *n* = 502 BDcfP sedated patients. Scatter dot plot depicting the average propofol 1% flow rate (ml/h) in correlation with the periprocedural mean arterial pressure, blue dots: without theodrenaline/cafedrine, red dots: with theodrenaline/cafedrine. The black line indicates simple linear regression with 95% confidence intervals of propofol flow rate in correlation to mean arterial pressure. Spearman correlation coefficient: *r* = 0.103 (*p* < 0.001). The dotted line indicates a critical mean arterial pressure of 60 mmHg. Of note, the perfusor used allows a maximum flow rate of 99 mL/h. However, calculatory flow rates may be above this level as boluses are included in the average flow rate.

Regarding blood pressure management, supportive theodrenaline plus cafedrine application was needed in 64 cases (12.5%). The use of theodrenaline plus cafedrine was significantly associated with a reduced mean arterial pressure (see Figure 2). While periprocedural mean blood pressure was 77 (±14) mmHg in the not-treated cohort, the mean blood pressure of the theodrenaline plus cafedrine-treated cohort was 60 (±12) mmHg (*p* < 0.001). Here, the use of theodrenaline plus cafedrine was significantly associated with the presence of cardiovascular comorbidities (*p* = 0.005): While 21.7% of the patients with cardiovascular comorbidities needed supportive treatment, only 10.5% in the non-comorbid group received theodrenaline plus cafedrine. Moreover, patients with arterial hypertension received theodrenaline plus cafedrine more often (i.e., 18.5% vs. 8.6%, *p* = 0.001). Other than that, COPD patients were likewise prone to this mild vasopressive therapy (i.e., 23.4% of the COPD patients vs. 10.9% of the non-comorbid ones, *p* = 0.008). Other than that, a general comorbidity severity represented by the ASA score did not associate with the use of mild vasopressive therapy (*p* = 0.664) and mean arterial pressure did not significantly differ between ASA 2, 3, and 4 (i.e., 74.4 ± 13.9 mmHg, 73.8 ± 13.8 mmHg, and 77.5 ± 13.9 mmHg, respectively, *p* = 0.295).

If compared to a propofol-based approach, the ketamine-based sedation resulted in significantly higher mean periprocedural blood pressure values (i.e., 97 [±30] mmHg in the ketamine-cohort vs. 74 [±14] mmHg in the propofol cohort, *p* = 0.028).

However, propofol flow rate (mL/h) did not correlate with mean arterial pressure during the intervention (see Figure 2, Spearman correlation coefficient: *r* = 0.103, *p* < 0.001).

### 4.5. Outcome

A total of 70 patients (13.6%) received interventional flexible bronchoscopy/EBUS-TBNA in an outpatient or day-care hospital setting. The other 443 patients were examined within an on-ward stay. A total of 311 patients (60.6%) stayed exactly one day and left the hospital afterward, the other 132 patients stayed longer due to therapeutic interventions. The median in-hospital stay was 1 (95% CI: 0.937–1.063) day and mean stay was 3.3 (±7.0) days.

No fatal complications occurred during interventional bronchoscopy. However, one 75-year-old female patient required resuscitation due to a malignant pericardial effusion, that was not considered hemodynamically relevant via echocardiography directly before the procedure. Here, during the flexible bronchoscopy, oxygen saturation, as well as blood pressure, dropped unresponsive to theodrenaline and cafedrine as well as fractioned 1 mg adrenaline 0.1% intravenously (far left red dot in Figure 2). After emergency drainage of the pericardium, vital signs consolidated and she was transferred to the intensive care unit, where she died two days after bronchoscopy from rapidly progressing lung cancer. Three further patients did not survive the in-hospital stay after but not due to flexible interventional bronchoscopy and EBUS-TBNA. One patient died due to pre-existing infectious complications resulting from long-term toxic bone marrow failure following chemotherapeutic intervention of a late recurrent embryonal carcinoma. Two other patients died within a week after diagnosis of rapidly progressive SCLC in an in-hospital palliative radio-chemotherapeutic treatment approach.

## 5. Discussion

Currently, sedation techniques for interventional bronchoscopy continue to be adopted from published gastrointestinal endoscopy sedation protocols [1,6]. However, during bronchoscopy, an inevitable but basically life-threatening airway obstruction might enhance risks for periprocedural morbidity and mortality. Here, we present retrospective real-world data on *n* = 502 patients that underwent flexible interventional bronchoscopy and EBUS-TBNA via a safe and feasible sedative approach of pethidine/meperidine combined with continuous flow propofol treatment (BDcfP) mainly provided by a single bronchoscopist and a well-trained and experienced endoscopy nurse due to personnel shortage.

Proof of concept of a continuous flow application of propofol resulted in higher absolute propofol doses with a prolonged duration of the intervention (see Figure 1). Additionally, small midazolam boluses (i.e., 1 mg to 2.5 mg bolus steps) were used as co-sedatives and propofol-saving agonists [12] (see Figure 1, red dots). However, midazolam was not correlated to a higher propofol dose or a longer duration of the procedure.

As expected, sedation based on ketamine plus midazolam in *n* = 10 cases was significantly less vasodilative than the pethidine/meperidine plus propofol-based approach (*p* = 0.026) and hence has to be considered in patients with a greater risk of hypotension [24]. In detail, pethidine/meperidine plus propofol reduced the mean blood pressure from 96 to 74 mmHg (i.e., −12 mmHg) (paired *t*-test *p* < 0.001), while ketamine plus midazolam held the mean blood pressure at 103 mmHg (i.e., ±0 mmHg) (paired *t*-test for ketamine plus midazolam pre-interventional vs. periprocedural, *p* = 0.987). Though ketamine is known for bronchodilation, it rarely might cause bronchial spasm [11] and hypersalivation [27,28] and hence can complicate examination conditions, especially in flexible interventional bronchoscopy and EBUS-TBNA. 

It is unquestionable, that examinations should be performed by bronchoscopists with sufficient experience in critical care medicine and on different sedative drugs, possible antidotes, and knowledge of airway and circulation management. Moreover, mild vasoconstrictive therapy with theodrenaline plus cafedrine is a safe treatment to encounter sedation-induced intermittent mild hypotension.

Here, via analysis of the use of theodrenaline plus cafedrine, we identified patients at risk for the development of periprocedural vasodilation. We found patients with arterial hypertension (*p* = 0.001), cardiovascular comorbidities (*p* = 0.005), and COPD (*p* = 0.008) at higher risk for hypotension (see Figure 2, red dots). On the contrary, higher propofol flow rates were not associated with hypotension (see Figure 2, linear regression line). For the present sedation protocol, these previously mentioned comorbidities seem to be more relevant in planning sedation and anticipating complications, than the ASA score.

With respect to outcome, solely patients with end-stage lung cancer disease or cancer treatment-related deficiencies presented intermediate- to short-term fatal outcome, that was not expedited by the bronchoscopic procedure itself.

The present data support the use of BDcfP as a safe and feasible sedation technique for flexible interventional bronchoscopy and EBUS-TBNA even in a limited personnel approach. Due to a lack of nursing care beds, 70 patients were examined and intervened in an outpatient or day-case hospital setting—as routinely performed e.g., in the UK [29]—and further 311 patients (i.e., in sum 74.3% of the analyzed patients) left the examination site within 24 h after the intervention. Still, full reimbursement of EBUS-TBNA following German diagnosis-related groups (G-DRG) requires an in-hospital stay for at least one night.

Our real-world retrospective study is limited by its single-center design and descriptive analysis. Moreover, we are fully aware that the presented personnel shortage might be critical. Undoubtedly, the rapid involvement of additional medical personnel should be ensured in case of an unexpected complication during the procedure. Due to turnover in staff, we have now implemented a permanent second endoscopy nurse at every diagnostic or interventional bronchoscopy.

Yet, by documenting safe and feasible sedation protocols and identifying risk factors for periprocedural complications, we can provide insight into state-of-art conditions of diagnostics and treatment, even for non-medical workers. Based upon these data, healthcare decision-makers and politicians should initiate larger and multi-centric proof-of-concept trials and possibly consider offering full reimbursement for flexible interventional bronchoscopy and EBUS-TBNA in low-risk patients in an outpatient setting under pre-defined safety and sedation measures including adequate staffing.

## Figures and Tables

**Table 1:** Patient characteristics of the cohort.

	*n* of Total (513)	In % of Total
Sex		
Female	188	36.6
Male	325	63.4
Age		
Mean age (±SD) (years)	60.5 (±13.6)	
Median age (Q1–Q3) (years)	63 (54–70)	
Comorbidities		
Cardiovascular	92	17.9
(a) Coronary artery disease	53	10.3
(b) Congestive heart failure	31	6.0
(c) Cerebral/peripheral obstructive artery disease	31	6.0
Pulmonary	99	19.3
(a) COPD	64	12.5
(b) Bronchial asthma	20	3.9
(c) Obstructive sleep apnea	17	3.3
Diabetes mellitus	65	12.7
Arterial hypertension	200	39.0
Renal insufficiency	36	7.0
ASA Score		
1	0	0.0
2	126	24.6
3	348	67.8
4	39	7.6
5	0	0.0
Average time until discharge		
Mean time (±SD) (days)	3.3 (±7.0)	
Median time (Q1–Q3) (days)	1 (1–2)	

SD—standard deviation; Q1–Q3—interquartile range; COPD—chronic obstructive pulmonary disease.

**Table 2:** Characteristics of the performed procedures and sedation protocol.

	*n* of Total (513)	In % of Total
Time of total procedure		
Mean time (±SD) (minutes)	92.2 (±29.6)	
Median time (Q1–Q3) (minutes)	90 (71–109)	
Time of interventional procedure		
Mean time (±SD) (minutes)	55.7 (±24.6)	
Median time (Q1–Q3) (minutes)	56 (37–73)	
Present trained bronchoscopists		
1	360	70.2
≥2	153	29.8
Analgosedative regime		
Propofol	502	97.9
Mean dose (±SD) (mg)	500.3 (±217.0)	
Median dose (Q1–Q3) (mg)	480 (370–565)	
Pethidine/Meperidine	493	96.1
Mean dose (±SD) (mg)	50.1 (±2.3)	
Median dose (Q1–Q3) (mg)	50 (50–50)	
Midazolam	48	9.4
Mean dose (±SD) (mg)	5.8 (±2.6)	
Median dose (Q1–Q3) (mg)	5 (5–5)	
Ketamine	12	2.3
Theodrenaline + Cafedrine	64	12.5
Norepinephrine	3	0.6
Epinephrine	1	0.2
Prednisone	30	5.8
Obtained specimen diagnosis		
NSCLC	110	21.4
SCLC/LCNEC	43	8.4
Cancer other than lung	52	10.1
Sarcoidosis	61	11.9
Rheumatic diseases	8	1.6
Other interstitial lung diseases	7	1.4
Infectious cause	13	2.5
Anthracosis	15	2.9
Negative for malignancy/inflammation	204	39.8

SD—standard deviation; Q_1_–Q_3_—interquartile range; NSCLC—non-small cell lung cancer; SCLC—small cell lung cancer; LCNEC—large cell neuroendocrine lung cancer.

**Table 3:** Characteristics of the performed interventions and complications.

	*n* of Total (513)	In % of Total
Performed interventions		
Endobronchial ultrasound (EBUS)	461	89.9
Bronchial washing	352	68.6
Bronchoalveolar lavage (BAL)	146	28.5
Cryo biopsy	111	21.6
Forceps biopsy	84	16.4
Brush cytology	62	12.1
Fluoroscopy	58	11.3
Argon plasma coagulation/high-frequency electricity	56	10.9
Navigation bronchoscopy	21	4.1
Complications		
Overall	95	18.5
Bleeding	70	13.6
(a) Minor bleeding	48	9.4
(b) Moderate bleeding	22	4.3
(c) Severe bleeding	0	0.0
Pneumothorax	0	0.0
Bronchial obstruction	33	6.4
Hypertension	3	0.6
Agitation	6	1.2
Others ^$^	2	0.4
	***n* of EBUS (461)**	**in % of *n* = 461**
Number of TBNA per patient		
Mean (±SD) (number of TBNA)	5.8 (± 3.1)	
Median (Q1–Q3) (number of TBNA)	6 (3–8)	
TBNA lymph node position		
7	257	55.7
2R	30	6.5
4R	109	23.6
10R	123	26.7
11R	127	27.5
12R	21	4.6
2L	12	2.6
4L	44	9.5
10L	57	12.4
11L	93	20.2
12L	9	2.0

SD—standard deviation; Q_1_–Q_3_—interquartile range; TBNA—transbronchial needle aspiration; ^$^ other complications—including *n* = 1 pericardial effusion [not hemodynamically relevant prior to bronchoscopy], *n* = 1 technically complicated fiberoptic intubation with intermittent mask ventilation.

**Table 4:** Vital parameters of *n* = 502 continuous flow propofol sedated patients.

	Pre-Interventional	Peri-Procedural	Post-Interventional	*p*-Value
Oxygen saturation (% SO_2_)				
Mean (±SD)	97.0 (±2.4)	95.8 (±5.4)	96.7 (±1.9)	* <0.001
				** <0.001
				*** 0.010
Median (Q1–Q3)	98 (96–98)	98 (95–99)	97 (96–98)	^§^ <0.001
Heart frequency (bpm)				
Mean (±SD)	78 (±16)	76 (±16)	75 (±14)	* 0.012
				** 0.003
				*** <0.001
Median (Q1–Q3)	76 (66–88)	76 (66–84)	75 (65–84)	^§^ <0.001
Systolic blood pressure (mmHg)				
Mean (±SD)	135 (±24)	98 (±21)	115 (±17)	* <0.001
				** <0.001
				*** <0.001
Median (Q1–Q3)	132 (117–148)	98 (85–107)	117 (105–127)	^§^ <0.001
Mean blood pressure (mmHg)				
Mean (±SD)	96 (±15)	74 (±14)	84 (±11)	* <0.001
				** <0.001
				*** <0.001
Median (Q1–Q3)	94 (86–104)	72 (65–81)	83 (76–90)	^§^ <0.001
Diastolic blood pressure (mmHg)				
Mean (±SD)	77 (±13)	62 (±12)	69 (±11)	* <0.001
				** <0.001
				*** <0.001
Median (Q1–Q3)	76 (67–81)	59 (56–67)	66 (65–76)	^§^ <0.001

SO_2_—Oxygen saturation; bpm—heart beats per minute; mmHg—millimeter of mercury; SD—standard deviation; Q1–Q3—interquartile range; ^§^ Friedman-Test; * paired *t*-test pre-interventional vs. interventional, ** paired *t*-test interventional vs. post-interventional, *** paired *t*-test pre-interventional vs. post-interventional.

## Data Availability

The data presented in this study are available on reasonable request from the corresponding author. These data are not publicly available due to ethical concern.
